# Characterization of ARVC substrate on MRI and electrophysiological mapping

**DOI:** 10.1186/1532-429X-18-S1-P197

**Published:** 2016-01-27

**Authors:** Stephanie Clement-Guinaudeau, Marjorie Salel, Olivier Corneloup, Gael Dournes, Michel Montaudon, François Laurent, Hubert Cochet

**Affiliations:** grid.42399.350000000405937118Hôpital Haut-Lévêque, Bordeaux, France

## Background

In patients with ARVC, breath-hold late gadolinium-enhanced imaging (LGE) may lack spatial resolution to assess the dysplasic substrate on the thin RV wall. We used electrophysiological contact mapping to evaluate the accuracy of breath-hold LGE (BH-LGE), free-breathing LGE (FB-LGE) and cine imaging in describing the extent of dysplasic substrate in ARVC.

## Methods

We studied consecutive patients with definite ARVC diagnosis according to Task Force criteria (TFC), undergoing electrophysiological study for ventricular tachycardia. CMR imaging was performed on a 1.5T system (Avanto, Siemens, Erlangen, Germany). SSFP cine imaging was performed in 2 stacks of contiguous 6 mm-thick slices encompassing the whole ventricles in short axis and 4-chamber orientations. BH-LGE imaging was performed 10 min after the injection of 0.2 mmol/Kg gadoterate meglumine using a 3D turbo FLASH sequence in 3 stacks of contiguous 6 mm-thick slices encompassing the whole ventricles in short axis, 2-chamber and 4-chamber orientations (pixel size 1.6 × 1.6 × 6 mm). FB-LGE acquisition was initiated 15 min after contrast using an inversion recovery-prepared and respiratory navigated 3D Turbo FLASH sequence with fat saturation, in order to acquire a whole heart volume at higher spatial resolution (pixel size 1.25 × 1.25 × 2.5mm). Wall motion abnormalities (WMA) and LGE were assessed by 2 observers analyzing the images in consensus. This substrate was distributed over a biventricular 16-segment model: the RV and LV free walls comprised 7 segments each (3 basal, 3 midventricular and 1 apical), and the septum comprised 2 segments (basal and midventricular). All patients underwent electrophysiological contact mapping during sinus rhythm on RV endocardium and RV and LV epicardium. Low bipolar voltage and local abnormal ventricular activity (LAVA) were distributed over the same segmentation.

## Results

23 patients were included (age 45 ± 14years, 5 women). MRI was positive for a major TFC in 14/23(61%), for a minor TFC in 3/23(13%), and it was negative in 6/23 patients (26%). RVEF and RVEDV were 38 ± 9% and 111 ± 24 mL/m^2^, respectively. LVEF and LVEDV were 57 ± 11% and 82 ± 18 mL/m^2^, respectively. On the RV, low voltage and/or LAVA were found at contact mapping in all patients, WMA in 22/23(96%), FB-LGE in 20/23(87%), and BH-LGE in 16/23(70%). On regional analysis, WMA and FB-LGE showed higher agreement with contact mapping than BH-LGE (k=0.72 and k=0.70 vs. k=0.55, respectively). On the LV, low voltage and/or LAVA were found at contact mapping in 13/23(57%) patients, WMA in 7/23(30%), FB-LGE in 13/23(57%) and BH-LGE in 13/23(57%). On regional analysis, FB- and BH-LGE showed higher agreement with contact mapping than WMA (k=0.77 and 0.77 vs. k=0.32).

## Conclusions

In patients with proven ARVC, cine imaging is more efficient in detecting dysplasic substrate than the conventional BH-LGE method on the RV, while FB-LGE imaging performs better. Conversely, LGE imaging is more efficient in detecting dysplasic substrate than cine imaging on the LV.

